# The extraocular muscle stem cell niche is resistant to ageing and disease

**DOI:** 10.3389/fnagi.2014.00328

**Published:** 2014-12-01

**Authors:** Luigi Formicola, Giovanna Marazzi, David A. Sassoon

**Affiliations:** UMRS 1166 INSERM, Stem Cells and Regenerative Medicine, Institute of Cardiometabolism and Nutrition (ICAN), University of Pierre and Marie Curie Paris VIParis, France

**Keywords:** extraocular muscles, muscle stem cell niche, Duchenne muscular dystrophy, PICs, satellite cells

## Abstract

Specific muscles are spared in many degenerative myopathies. Most notably, the extraocular muscles (EOMs) do not show clinical signs of late stage myopathies including the accumulation of fibrosis and fat. It has been proposed that an altered stem cell niche underlies the resistance of EOMs in these pathologies, however, to date, no reports have provided a detailed characterization of the EOM stem cell niche. PW1/Peg3 is expressed in progenitor cells in all adult tissues including satellite cells and a subset of interstitial non-satellite cell progenitors in muscle. These PW1-positive interstitial cells (PICs) include a fibroadipogenic progenitor population (FAP) that give rise to fat and fibrosis in late stage myopathies. PICs/FAPs are mobilized following injury and FAPs exert a promyogenic role upon myoblasts *in vitro* but require the presence of a minimal population of satellite cells *in vivo*. We and others recently described that FAPs express promyogenic factors while satellite cells express antimyogenic factors suggesting that PICs/FAPs act as support niche cells in skeletal muscle through paracrine interactions. We analyzed the EOM stem cell niche in young adult and aged wild-type mice and found that the balance between PICs and satellite cells within the EOM stem cell niche is maintained throughout life. Moreover, in the adult *mdx* mouse model for Duchenne muscular dystrophy (DMD), the EOM stem cell niche is unperturbed compared to normal mice, in contrast to *Tibialis Anterior* (TA) muscle, which displays signs of ongoing degeneration/regeneration. Regenerating *mdx* TA shows increased levels of both PICs and satellite cells, comparable to normal unaffected EOMs. We propose that the increase in PICs that we observe in normal EOMs contributes to preserving the integrity of the myofibers and satellite cells. Our data suggest that molecular cues regulating muscle regeneration are intrinsic properties of EOMs.

## Introduction

Duchenne muscular dystrophy (DMD) is the most common X-linked recessive disease in humans, affecting 1 in 3500 males and causing premature death in the second decade of life following cardiac and pulmonary failure (Tabebordbar et al., 2013). Duchenne muscular dystrophy is due to mutations in the dystrophin gene, which encodes a structural protein linking the cytoskeleton of the myofiber to the surrounding basal lamina (Ervasti and Campbell, 1991; Chaturvedi et al., 2001). The absence of functional dystrophin leads to degeneration of the myofibers, which results in repeated rounds of degeneration and regeneration (Wallace and McNally, 2009). Despite the regenerative response, muscle tissue gradually becomes replaced by fibrotic and fat tissue followed by loss of muscle function (Wallace and McNally, 2009; Tabebordbar et al., 2013). Satellite cells are the principal myogenic progenitor population in skeletal muscle that give rise to new myofibers (Relaix and Zammit, 2012). Satellite cells actively proliferate during prenatal life and progressively enter a quiescent state after birth (Bismuth and Relaix, 2010). In the adult, satellite cells remain quiescent and can be identified based upon their location under the basal lamina of the myofibers as well as by the expression of *Pax7* (Sambasivan and Tajbakhsh, 2007; Pallafacchina et al., 2010; Pannérec et al., 2013). While quiescent in the adult, satellite cells re-enter the cell cycle in response to injury to give rise to new myofibers as well as restore the satellite cell pool (Bismuth and Relaix, 2010; Yin et al., 2013). Muscle tissue also possesses multiple interstitial cell populations that regulate satellite cell function (Pannérec et al., 2012; Relaix and Zammit, 2012). The fibroadipogenic progenitors (FAPs) that reside in the interstitium are required for proper regeneration (Pannérec et al., 2012; Yin et al., 2013). Fibroadipogenic progenitors become activated in response to injury and promote satellite cell differentiation *in vitro* (Joe et al., 2010; Uezumi et al., 2010). However, when satellite cells are depleted or functionally impaired, FAPs differentiate into adipocytes and contribute to fibrosis (Joe et al., 2010; Uezumi et al., 2010, 2011). We reported previously that the cell stress-mediator gene, *PW1/Peg3*, is expressed in multiple progenitor populations in adult tissues, including skeletal muscle (Mitchell et al., 2010; Besson et al., 2011). In skeletal muscle PW1/Peg3 expression is observed in satellite cells and a subset of interstitial cells referred to as positive interstitial cells (PICs; Mitchell et al., 2010; Pannérec et al., 2013). We demonstrated that PICs include the entirety of the FAPs and that this subpopulation expresses follistatin (FST) and insulin-like growth factor-1 (IGF-1) that account, in part, for their promyogenic activity (Pannérec et al., 2013; Formicola et al., under review and see Mozzetta et al., 2013). The close proximity of the PICs to the satellite cells suggests that they act in part as progenitor niche cells (Mitchell et al., 2010; Formicola et al., under review). Taken together, both the satellite cells and a subset of interstitial cells are required for proper muscle regeneration and impairment of either cell type can lead to loss of muscle tissue and increased fat and fibrosis which is typical of mid- to late-stage degenerative muscle diseases (Serrano et al., 2011; Shadrach and Wagers, 2011; Tabebordbar et al., 2013; Yin et al., 2013).

All skeletal muscle tissue is composed of the same basic cellular components, however specific groups of muscles display either increased sensitivity or resistance to muscle diseases. While the limb and diaphragm muscles are severely affected in most muscle myopathies, the extraocular muscles (EOMs) do not undergo a significant loss of function and maintain their tissue integrity in many myopathies including DMD and amyotrophic lateral sclerosis, whereas they are selectively targeted by some myopathies that do not affect other skeletal muscles such as the oculopharyngeal muscular dystrophy and myasthenia gravis (Kaminski et al., 1992; Khurana et al., 1995; Porter et al., 1995; Davies et al., 2006; Abu-Baker and Rouleau, 2007; Soltys et al., 2008). The EOMs consist of six muscles surrounding the ocular globe responsible for eye movements (Bohnsack et al., 2011). These muscles have a distinct embryological origin compared to other skeletal muscles of the body and display unique properties including the expression of a specific myosin heavy chain isoform (Porter et al., 1995, 2001, 2006; Porter, 2002). Moreover, EOM satellite cells show a different genetic requirement during embryonic development as well as postnatal differentiation (Sambasivan et al., 2009, 2011). Following engraftment into limb muscles, EOM satellite cells efficiently differentiate into myofibers and form myofibers that do not express EOM-specific myosin isoforms indicating that local niche-derived or nerve-specific signals are important in specifying muscle phenotype (Sambasivan et al., 2009). Several studies have described the presence of activated satellite cells in uninjured normal adult EOMs from different species (rabbit, mouse, monkey) (McLoon and Wirtschafter, 2002a,b, 2003; McLoon et al., 2004) as well as more satellite cells per myofiber as compared to limb muscles (McLoon et al., 2007), leading to the hypothesis that EOMs undergo continuous myonuclei addition providing a cellular basis for continued tissue remodeling throughout life. Other studies comparing adult EOMs and limb muscles revealed different transcriptomes and proteomes (Porter et al., 2001, 2003, 2006; Khanna et al., 2003, 2004; Pacheco-Pinedo et al., 2009; Lewis and Ohlendieck, 2010). Interestingly, members regulating the transforming growth factor beta (TGFβ) and IGF-1 signaling pathways are differentially expressed between EOMs and limb muscles suggesting that the EOM progenitor cells are exposed to a more promyogenic environment (Porter et al., 2003; Pacheco-Pinedo et al., 2009). We have shown previously that PW1/Peg3 is expressed by muscle niche cells (Besson et al., 2011; Pannérec et al., 2012, 2013) that express several of these promyogenic factors in limb muscles and that the niche has a profound influence on regenerative capacity (Mozzetta et al., 2013; Formicola et al., under review). Since the EOM is resistant to multiple myopathies, it is possible that the EOM niche differs substantially from other muscle groups.

In this study, we compared EOMs to limb muscles in normal adult and aged mice as well *mdx* mutant mice. While EOMs have the same number of satellite cells per fiber as compared to limb muscles, we note that the number of PICs is markedly higher. Limb muscle derived PICs secrete both IGF-1 and FST (Formicola et al., under review), and here we observed a higher level of these growth factors in EOMs. Furthermore, while both EOMs and limb muscles display a decline in satellite cell number with age, PICs are maintained in EOMs at a similar ratio with satellite cells at all ages whereas they are markedly decreased in limb muscles with age. Moreover, PICs are maintained at higher numbers in *mdx* limb muscles as compared to wild-type counterparts and these high numbers are comparable to the ones observed in wild-type EOMs. Taken together, these data reveal that the PIC population is uniquely regulated in EOMs and suggest that the maintenance of a high number of PICs provides a more promyogenic environment. This unique stem cell niche may contribute to EOM resistance to multiple muscle degenerative diseases and age-related functional decline through the maintenance of tissue plasticity throughout life.

## Methods

### Mice

Animal models used were: 7 week-old and 18 month-old C57Bl6J mice, 7 week-old and 18 month-old C57Bl6J PW1IRESnLacZ transgenic reporter mice (PW1^nlacZ^) (Besson et al., 2011), 7 week-old C57Bl10 and *mdx* (Bulfield et al., 1984) mice. All work with mice was carried out in adherence to French government guidelines.

### Histological analyses

*Tibialis Anterior* (TA) muscles were removed, mounted in tragacanth gum (Sigma Aldrich) and snap frozen in liquid nitrogen-cooled isopentane (Sigma Aldrich) as previously described (Mitchell et al., 2010). For EOM dissection, the skin of the head was removed to expose the eye. An incision of the basal part of the eyelids was performed and the globe was gently pulled out of the ocular cavity. A perpendicular cut in proximity of the skull inside the cavity was performed to release the globe with the EOMs attached *in situ*. Eyelids were removed from the globe, which was then mounted in tragacanth gum (Sigma Aldrich) and snap frozen in liquid nitrogen-cooled isopentane (Sigma Aldrich) as previously described (Mitchell et al., 2010). Muscles were cryosectioned (5–7 µm) before processing.

To stain nuclei and muscle fibers, cryosections were stained with hematoxilin and eosin (H&E) (Sigma Aldrich). For PW1^nLacZ^ mice, cryosections were stained with Xgal as previously reported (Besson et al., 2011) and Nuclear Fast Red solution (Sigma Aldrich) according to manufacturer’s instructions.

For immunofluorescence, EOMs and TA cryosections were fixed in 4% (w/v) paraformaldehyhede and processed for immunostaining as described previously (Mitchell et al., 2010). Primary antibodies used were: PW1 (Relaix et al., 1996) (rabbit, 1:3000), Pax7 (mouse, Developmental Studies Hybridoma Bank, 1:10), MyoD (mouse, BD Biosciences, 1:100), Ki67 (mouse, BD Biosciences, 1:100), Ki67 (rabbit, Abcam, 1:100), PH3 (rabbit, Abcam, 1:100), laminin (rabbit, Sigma, 1:100). Antibody binding was revealed using species-specific secondary antibodies coupled to Alexa Fluor 488 (Life Technologies), Cy3 or Cy5 (Jackson Immunoresearch). Nuclei were counterstained with DAPI (Sigma Aldrich).

### RNA extraction and qPCR

Extraocular muscles from six different C57Bl6J mice were pooled into a single sample. Tibialis Anteriors from three different C57Bl6J mice were analyzed separately. RNA extracts were prepared using RNeasy Fibrous Midi Kit (Qiagen) according to manufacturer’s instructions, and reverse transcribed using the SuperScript First-Strand Synthesis System (Life Technologies). Quantitative polymerase chain reaction was performed using SYBR^®^ green (Thermo Fisher Scientific) under the following cycling conditions: 95°C for 5 min followed by 50 cycles of amplification (95°C for 15 s, 61°C for 15 s and 72°C for 20 s), then 95°C for 5 s followed by a final incubation at 65°C for 1 min. Each sample was analyzed in triplicate. Primers sequences used were: FST, FWD 5′-CCCCAACTGCATCCCTTGTAAA-3′ and REV 5′-TCCAGGTGATGTTGGAACAGTC-3′; IGF-1, FWD 5′-TGCTCTTCAGTTCGTGTG-3′ and REV 5′-ACATCTCCAGTCTCCTCAG-3′; myostatin (MST), FWD 5′-GGCTCAAACAGCCTGAATCCAA-3′ and REV 5′-CCAGTCCCATCCAAAGGCTTCAAA-3′; 18S: FWD 5′-CGGCTACCACATCCAAGGAA-3′ and REV 5′-TATACGCTATTGGAGCTGGAA-3′. Levels of FST, IGF-1 and MST expression were normalized using 18S gene expression.

### Statistical analysis

All statistics were performed using an unpaired Student’s *t*-test in the StatView software. Values represent the mean ± s.e.m. ^*^p < 0.05, ^**^p < 0.01 and ^***^p < 0.001.

## Results

### EOM stem cell niche is conserved throughout postnatal life

**Figure 1:**
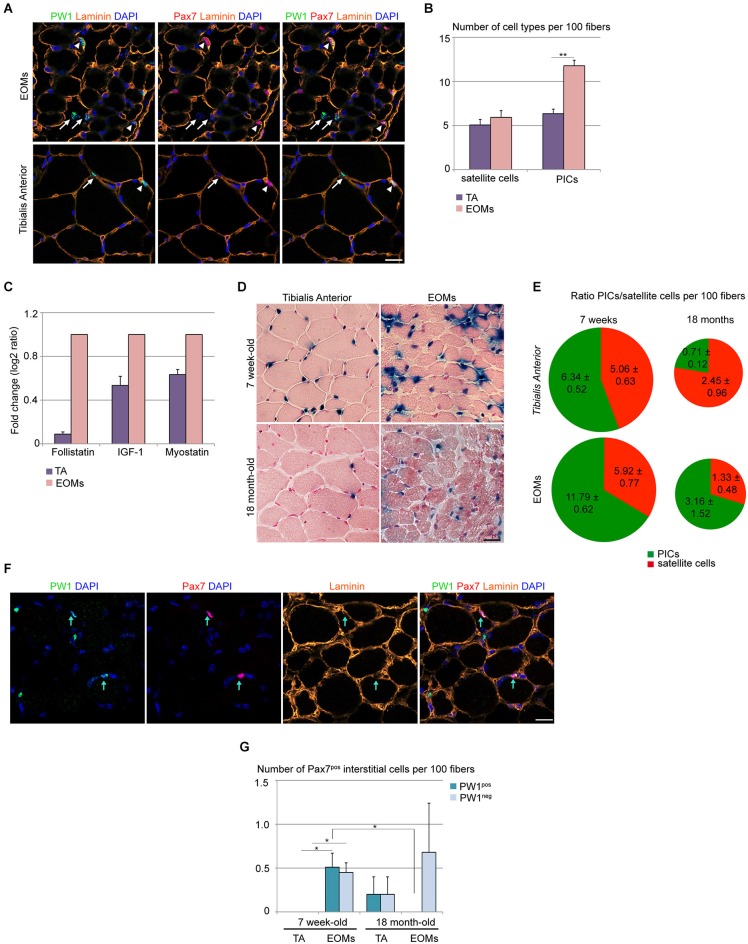
**Wild-type EOM stem cell niche is intrinsically different from limb muscles**. **(A)** Cross-sections of 7-week old EOMs (upper panels) and TA (lower panels) from C57Bl6 mice stained for PW1 (green) and the satellite cell marker Pax7 (red). Laminin staining (orange) shows the basal lamina. Nuclei were counterstained by DAPI (blue). Arrows indicate PICs, arrowheads indicate satellite cells. Scale bar, 50 µm. **(B)** Number of satellite cells and PICs per 100 fibers in 7-week old EOMs and TA cross-sections as stained in **(A)** revealed a bigger amount of PICs but not satellite cells in EOMs compared to TA. **(C)** Fold change of FST, IGF-1 and myostatin expression levels from qPCR analysis on total RNA extracts from EOMs and TA from 7 week-old wild-type mice revealed an strong increase in FST expression in EOMs. For TA muscles, three different animals (*n* = 3) were considered separately; for EOMs, six different animals (*n* = 6) were pooled into one sample. Each sample was analyzed in duplicate. Error bar indicates s.e.m. calculated for number of samples. **(D)** Xgal staining on cross-sections of 7-week old (upper panels) and 18 month-old (lower panels) EOMs and TA from PW1^nLacZ^ mice. Nuclei and myofibers were counterstained with Nuclear Fast Red™ Solution. Scale bar, 40 µm. **(E)** Ratio between PICs (green) and satellite cells (red) per 100 fibers in 7-week old and 18-month old TA and EOMs cross-sections demonstrated that EOM but not TA stem cell niche is retained throughout life. **(F)** Cross-sections of 7-week old EOMs stained as in **(A)**. We observe Pax7^pos^ cells totally or partially surrounded by the basal lamina and often co-expressing PW1. Arrows indicate double-labeled PW1^pos^Pax7^pos^ interstitial cells. **(G)** Number of Pax7^pos^ interstitial cells (PW1^pos^ and PW1^neg^ subsets) per 100 fibers in 7 week-old and 18 month-old EOMs and TA cross-sections stained as in **panel A**. For all graphs, values represent the mean number of cells ± s.e.m. For **(B,E)**, PICs were determined as interstitial PW1^pos^Pax7^neg^ cells, satellite cells were determined as Pax7^pos^ cells underneath the basal lamina. Statistical significance was calculated from at least three animals of each condition. *p < 0.05 **p < 0.01 ***p < 0.001.

It has been reported previously that *PW1/Peg3* RNA levels are higher in normal EOMs as compared to limb muscles (Porter et al., 2003), suggesting either an increase of *PW1* gene expression or an increase of the total number of *PW1*-expressing cells in EOMs. We therefore analyzed the EOM and limb muscle progenitor cell niche and compared our results with those obtained previously for limb muscles during postnatal stages (Mitchell et al., 2010; Pannérec et al., 2013). We have shown previously that in limb muscles PICs and satellite cells undergo a progressive decline within the first 3 weeks after birth, however these two cell types maintain a 1:1 ratio in homeostatic conditions in young and adult mice (Mitchell et al., 2010; Formicola et al., under review). We analyzed 7 week-old wild-type EOMs and TA muscles and found a 2-fold higher number of PICs in EOMs as compared to TA, whereas the number of satellite cells per muscle fiber was the same in the two sets of muscles (Figures 1A,B). This higher number of PICs per muscle fiber results in a PICs/satellite cells ratio of 2:1 in EOMs as compared to 1:1 found in the TA (Figure 1B; Mitchell et al., 2010; Formicola et al., under review). We (Mozzetta et al., 2013; Formicola et al., under review) described recently that PICs express promyogenic factors, such as FST and IGF-1, which are able to counteract the antimyogenic effect exerted by MST and other TGFβ superfamily members, including activins (Amthor et al., 2004; Latres et al., 2005; Lee et al., 2010). We wondered whether the larger PIC population observed in EOMs could provide a higher level of promyogenic factors in the EOM. Our analyses of whole EOM extracts revealed that the EOMs display levels of expression of *Fst* and *Igf-1* that are respectively 11 and 2 folds higher as compared to the TA, whereas differences in *Mst* levels are less pronounced (1.5 fold higher level of expression in EOMs as compared to TA; Figure 1C). Using PW1^nLaz^ reporter mouse (Besson et al., 2011) we then compared EOMs and TA from 7 week-old and 18 month-old mice. We noted that while TA shows a marked decrease in PW1-expressing cells with age, in EOMs the amount of PW1-expressing cells were less affected (Figure 1D). A more detailed analysis revealed that although both muscle groups show a decrease in PICs and satellite cell number with age, PICs/satellite cells ratio remains unchanged in aged EOMs as compared to young EOMs (Figure 1E). Specifically, PICs from limb muscles undergo a marked decline as compared to satellite cells, as aged TA exhibits a 0.3:1 ratio between PICs and satellite cells (Figure 1E), supporting the notion that factors regulating progenitor cells within the niche are different in EOMs as compared to limb muscles. In addition, we noted a rare population of interstitial cells that expressed Pax7 in 7 week-old EOMs, completely or partially surrounded by the basal lamina (Figure 1F), whereas we did not observe any Pax7^pos^ interstitial cell in age-matched TA (Figures 1F,G), suggesting that a subpopulation of satellite-like cells has a different anatomical location in EOMs as compared to limb muscles. Moreover, we noted that half of the Pax7^pos^ interstitial cells observed in adult EOMs co-express PW1 (Figure 1G), raising the possibility that a subset of PICs is committed to the myogenic lineage. We note that Pax7^pos^ interstitial cells can also be detected in aged limb muscles as well as aged EOMs (Figure 1G). A previous report showed that a subset of aged limb muscle satellite cells is more prone to exit quiescence due to high homeostatic FGF-2 expression (Chakkalakal et al., 2012). Interestingly, FGF-2 levels have been reported to be elevated in human EOMs as compared to limb muscles (Fischer et al., 2005). Whether these Pax7^pos^ interstitial cells are a subpopulation of FGF-2-responsive PICs that enter the myogenic lineage through expression of Pax7 or they are a subset of satellite cells responding to FGF-2 with a different anatomical location is an issue that remains to be resolved.

In order to know if these differences in EOMs are related to a different activation status of progenitor cells in EOMs as compared to TA, we checked for cell cycle markers (as Ki67 and PH3) as well as myogenic activation marker MyoD. We failed to detect expression of Ki67 and PH3 as well as MyoD in PICs or satellite cells from EOMs indicating that PICs and satellite cells are not activated in EOMs (data not shown) and consistent with observations in TA and other skeletal muscles (Pallafacchina et al., 2010) but in contrast with previous reports in EOMs (McLoon and Wirtschafter, 2002a,b, 2003; McLoon et al., 2004). This likely reflects the different animal species and ages considered (rabbits, mice, rats) as well as the different techniques used to reveal satellite cell activation and proliferation (i.e., single myofiber reconstruction vs. histological analysis of transversal sections) (McLoon and Wirtschafter, 2002a,b; McLoon et al., 2004). Moreover, in those previous reports, no direct co-labeling of MyoD with satellite cell markers was performed and cell proliferation status was assessed through BrdU incorporation (McLoon and Wirtschafter, 2002a,b; McLoon et al., 2004). Nonetheless, the presence of both MyoD^pos^ cells and BrdU-labeled cells were rare events (McLoon and Wirtschafter, 2002a,b; McLoon et al., 2004). We noted that PW1^pos^Pax7^pos^ interstitial cells decline with age in EOMs, however we still detected PW1^neg^Pax7^pos^ interstitial cells (Figure 1G). The role and biological significance of these two subsets of the Pax7^pos^ interstitial cell population based on PW1 expression remains to be determined. Taken together, our observations strongly support the notion that the EOM stem cell niche composition is intrinsically different from that seen in limb muscles.

### *Mdx* mice display an unaltered EOM stem cell niche

**Figure 2:**
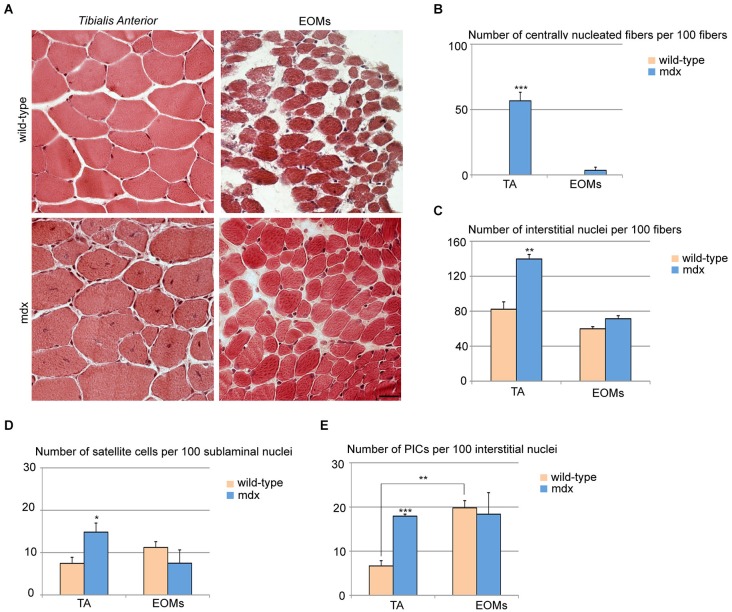
**Extraocular muscles from *mdx* and wild-type mice exhibit a similar muscle stem cell niche**. **(A)** Cross-sections of 7-week old TA (upper panels) and EOMs (lower panels) from *mdx* and age-matched wild-type mice stained with hematoxylin and eosin showed large regenerating areas in TA but not EOMs from *mdx* mice. **(B,C)** Number of centrally nucleated fibers **(B)** and interstitial nuclei **(C)** per 100 fibers indicated the presence of an ongoing regeneration process in *mdx* TA but not EOMs. **(D,E)** Number of satellite cells per 100 sublaminal nuclei **(D)** and PICs per 100 interstitial nuclei **(E)** revealed an activation of both progenitor cell types in TA but not EOMs from *mdx* mice, as compared to their wild-type counterparts. Positive interstitial cells were determined as interstitial PW1^pos^Pax7^neg^ cells, satellite cells were determined as Pax7^pos^ cells underneath the basal lamina. For all graphs, values represent the mean number ± s.e.m. Statistical significance was calculated from at least three animals per each condition. *p < 0.05 **p < 0.01 ***p < 0.001.

*Mdx* skeletal muscles undergo continuous cycles of degeneration and regeneration due to a defect in the dystrophin gene, making it a valuable model for the study of DMD (Burghes et al., 1987; Partridge, 2013; Tabebordbar et al., 2013). While the mice and humans vary in the degree of disease severity, both share the feature that the EOMs are not affected (Kaminski et al., 1992; Porter et al., 1995, 2003). We analyzed the EOM and TA stem cell niches in 7 week-old wild-type and *mdx* mice. EOM cross-sections from *mdx* mice did not display histological signs of ongoing regeneration and looked as their wild-type counterparts, whereas TA cross-sections showed widespread regions with centrally nucleated fibers, as previously reported (Wallace and McNally, 2009; Figures 2A,B). We observed a single and highly restricted region containing a few centrally nucleated fibers in one EOM in only one out of three mice examined. Furthermore, we observed an increase in the number of interstitial cells in *mdx* TA as compared to wild-type TA, but no changes in EOMs (Figure 2C), confirming that *mdx* EOMs were unperturbed. Both satellite cells and PICs were increased in *mdx* TA as compared to its wild-type counterpart (Figures 2D,E), whereas *mdx* EOMs displayed an unchanged content of PICs and satellite cells as compared to wild-type (Figures 2D,E). Taken together, these data support the hypothesis that EOM stem cell niche is unperturbed in *mdx* as compared to wild-type. Interestingly, the number of PICs in *mdx* TA is comparable to wild-type EOMs (Figure 2E). Based on previous observations strongly indicating a role of PICs/FAPs as support niche cells, our data suggest that mechanisms normally occurring during muscle regeneration to promote progenitor cell survival, activation and differentiation, are intrinsic to the EOM stem cell niche.

## Discussion

Duchenne muscular dystrophy is the most common form of muscular dystrophy in humans affecting boys, leading to a loss of skeletal muscle mass and function and premature death following heart and respiratory failure (Wallace and McNally, 2009; Shadrach and Wagers, 2011; Tabebordbar et al., 2013). In late-stage DMD, muscle fibers are replaced by fibrotic and fat tissue, due to a massive deregulation of signaling pathways within the muscle tissue and a promotion of fibrosis (Wallace and McNally, 2009; Serrano et al., 2011; Tabebordbar et al., 2013). Several observations indicate that FAPs are the main source of fibrotic extracellular matrix deposition and adipocytes in this context (Joe et al., 2010; Uezumi et al., 2010, 2011; Pannérec et al., 2013). Nonetheless, FAPs are important regulators of the muscle regeneration process (Joe et al., 2010; Uezumi et al., 2010), suggesting a dual role for these cells in governing muscle homeostasis as well as the importance of the microenvironment in directing their behavior. The observations that human as well as mouse EOMs are resistant to several dystrophies including DMD as compared to other sets of skeletal muscles of the body (Porter et al., 1995; Porter, 2002) suggest that muscle-type specific endogenous mechanisms operate in the stem cell niche leading to a more efficient regenerative response or protection against fiber atrophy or degeneration. We recently proposed that PICs, which include the FAP population, act as support niche cells for satellite cells in postnatal skeletal muscle by secreting promyogenic factors (Pannérec et al., 2013; Formicola et al., under review). Our data confirm previous observations showing that the EOM stem cell niche is intrinsically different as compared to limb muscles (Porter et al., 2001, 2003; Porter, 2002; Khanna et al., 2003). Adult EOMs display a larger PIC population and an increased expression of the promyogenic factors FST and IGF-1, in contrast to limb muscles where PICs and satellite cells are in equal amounts (1:1) during postnatal growth and adulthood (Mitchell et al., 2010; Formicola et al., under review). Interestingly, despite a decline in both PICs and satellite cells in aged muscle, the ratio between these two progenitor cell types is tightly conserved in EOMs whereas the PIC compartment undergoes a marked decline with age in limb muscles. We propose that the EOM provides a more promyogenic environment as compared to limb muscle that is more resistant to diseases throughout life. We note that a recent study described the presence of fibroadipogenic Sca1^pos^ progenitor cells in the periocular connective tissue that have a Myf5^pos^Pax3^pos^ origin (Stuelsatz et al., 2014b). While we previously demonstrated that limb-isolated PICs are not derived from Pax3^pos^ progenitor cells (Mitchell et al., 2010), a possible different developmental origin of EOM PICs should not be discarded and could account for the different features exhibited by the muscle stem cell niche in normal as well as *mdx* and aged EOMs as compared to the limb muscles. Moreover, our observations of aged muscles are in contrast with hypotheses that an increase in FAP number in aged muscles accounts for an increase in fibrosis during sarcopenia (Shadrach and Wagers, 2011; García-Prat et al., 2013) and strongly support the idea that the balance of interactions between the niche components is the major determinant of muscle homeostasis rather than single cell population levels. Indeed, we found that EOM stem cell niche from *mdx* mice do not display any particular difference as compared to wild-type mice and *mdx* EOMs are not affected by the disease. It is interesting to note that the regeneration process in *mdx* limb muscles induces an increase of satellite cells and PICs up to a level comparable to the one observed in wild-type EOMs. A previous report has described increased *Fst* and *Mst* gene expression levels in limb muscles from young *mdx* mice as compared to wild-type counterparts (Abe et al., 2009) and we observed in this study a similar trend in wild-type EOMs as compared to wild-type limb muscles. We described recently that satellite cells secrete MST whereas PICs secrete FST (Mozzetta et al., 2013; Formicola et al., under review). The observations and data presented in this study support the hypothesis that changes in the stem cell niche composition and their signaling during muscle regeneration are intrinsic properties of EOMs at the steady state. Indeed, another study (Porter et al., 2003) showed that wild-type EOMs and *mdx* limb muscles highly express specific genes involved in muscle regeneration and fibrosis, such as interleukin-10 receptor beta (IL10Rβ), connective tissue growth factor (Ctgf), follistatin-like 1 (Fstl-1) and TGFβ-induced protein (TGFβi; Porter et al., 2003; Serrano et al., 2011; Deng et al., 2012). Interestingly, data from our previously published microarray comparing PICs and satellite cells reveal that these genes are upregulated in PICs (Pannérec et al., 2013). Moreover, a recent study revealed that satellite cells isolated from *mdx* EOMs as well as normal adult and aged EOMs exhibit a robust growth and self-renewal capacity *in vitro* and high engraftment performance as compared to their limb and diaphragm counterparts (Stuelsatz et al., 2014a). We propose that the EOMs are spared in degenerative diseases because they have a high number of PICs. This increase provides an environment rich in promyogenic and hypertrophic factors, which protect both the fibers and the satellite cells, preventing the loss of regenerative capacity observed in late stages of muscular dystrophies.

## Authors’ contributions

Luigi Formicola performed experiments. All authors designed experiments, analyzed and interpreted data and prepared the manuscript.

## Conflict of interest statement

The authors declare that the research was conducted in the absence of any commercial or financial relationships that could be construed as a potential conflict of interest.

## References

[B1] Abe S. Soejima M. Iwanuma O. Saka H. Matsunaga S. Sakiyama K.. (2009). Expression of myostatin and follistatin in Mdx mice, an animal model for muscular dystrophy. Zoolog. Sci. 26, 315–320. 10.2108/zsj.26.315 19715499

[B2] Abu-Baker A. Rouleau G. A. (2007). Oculopharyngeal muscular dystrophy: recent advances in the understanding of the molecular pathogenic mechanisms and treatment strategies. Biochim. Biophys. Acta 1772, 173–185. 10.1016/j.bbadis.2006.10.003 17110089

[B3] Amthor H. Nicholas G. McKinnell I. Kemp C. F. Sharma M. Kambadur R.. (2004). Follistatin complexes Myostatin and antagonises Myostatin-mediated inhibition of myogenesis. Dev. Biol. 270, 19–30. 10.1016/s0012-1606(04)00118-6 15136138

[B4] Besson V. Smeriglio P. Wegener A. Relaix F. Nait Oumesmar B. Sassoon D. A.. (2011). PW1 gene/paternally expressed gene 3 (PW1/Peg3) identifies multiple adult stem and progenitor cell populations. Proc. Natl. Acad. Sci. U S A 108, 11470–11475. 10.1073/pnas.1103873108 21709251 PMC3136256

[B5] Bismuth K. Relaix F. (2010). Genetic regulation of skeletal muscle development. Exp. Cell Res. 316, 3081–3086. 10.1016/j.yexcr.2010.08.018 20828559

[B6] Bohnsack B. L. Gallina D. Thompson H. Kasprick D. S. Lucarelli M. J. Dootz G.. (2011). Development of extraocular muscles requires early signals from periocular neural crest and the developing eye. Arch. Ophthalmol. 129, 1030–1041. 10.1001/archophthalmol.2011.75 21482859 PMC3248700

[B7] Bulfield G. Siller W. G. Wight P. A. Moore K. J. (1984). X chromosome-linked muscular dystrophy (mdx) in the mouse. Proc. Natl. Acad. Sci. U S A 81, 1189–1192. 10.1073/pnas.81.4.1189 6583703 PMC344791

[B8] Burghes A. H. Logan C. Hu X. Belfall B. Worton R. G. Ray P. N. (1987). A cDNA clone from the Duchenne/Becker muscular dystrophy gene. Nature 328, 434–437. 10.1038/328434a0 3614347

[B9] Chakkalakal J. V. Jones K. M. Basson M. A. Brack A. S. (2012). The aged niche disrupts muscle stem cell quiescence. Nature 490, 355–360. 10.1038/nature11438 23023126 PMC3605795

[B10] Chaturvedi L. S. Mukherjee M. Srivastava S. Mittal R. D. Mittal B. (2001). Point mutation and polymorphism in Duchenne/Becker muscular dystrophy (D/BMD) patients. Exp. Mol. Med. 33, 251–256. 10.1038/emm.2001.41 11795488

[B11] Davies J. E. Berger Z. Rubinsztein D. C. (2006). Oculopharyngeal muscular dystrophy: potential therapies for an aggregate-associated disorder. Int. J. Biochem. Cell Biol. 38, 1457–1462. 10.1016/j.biocel.2006.01.016 16530457

[B12] Deng B. Wehling-Henricks M. Villalta S. A. Wang Y. Tidball J. G. (2012). IL-10 triggers changes in macrophage phenotype that promote muscle growth and regeneration. J. Immunol. 189, 3669–3680. 10.4049/jimmunol.1103180 22933625 PMC3448810

[B13] Ervasti J. M. Campbell K. P. (1991). Membrane organization of the dystrophin-glycoprotein complex. Cell 66, 1121–1131. 10.1016/0092-8674(91)90035-W 1913804

[B14] Fischer M. D. Budak M. T. Bakay M. Gorospe J. R. Kjellgren D. Pedrosa-Domellöf F.. (2005). Definition of the unique human extraocular muscle allotype by expression profiling. Physiol. Genomics 22, 283–291. 10.1152/physiolgenomics.00158.2004 15855387

[B15] García-Prat L. Sousa-Victor P. Muñoz-Cánoves P. (2013). Functional dysregulation of stem cells during aging: a focus on skeletal muscle stem cells. FEBS J. 280, 4051–4062. 10.1111/febs.12221 23452120

[B16] Joe A. W. Yi L. Natarajan A. Le Grand F. So L. Wang J.. (2010). Muscle injury activates resident fibro/adipogenic progenitors that facilitate myogenesis. Nat. Cell Biol. 12, 153–163. 10.1038/ncb2015 20081841 PMC4580288

[B17] Kaminski H. J. al-Hakim M. Leigh R. J. Katirji M. B. Ruff R. L. (1992). Extraocular muscles are spared in advanced Duchenne dystrophy. Ann. Neurol. 32, 586–588. 10.1002/ana.410320418 1456746

[B18] Khanna S. Cheng G. Gong B. Mustari M. J. Porter J. D. (2004). Genome-wide transcriptional profiles are consistent with functional specialization of the extraocular muscle layers. Invest. Ophthalmol. Vis. Sci. 45, 3055–3066. 10.1167/iovs.03-1385 15326121

[B19] Khanna S. Merriam A. P. Gong B. Leahy P. Porter J. D. (2003). Comprehensive expression profiling by muscle tissue class and identification of the molecular niche of extraocular muscle. FASEB J. 17, 1370–1372. 12832294 10.1096/fj.02-1108fje

[B20] Khurana T. S. Prendergast R. A. Alameddine H. S. Tomé F. M. Fardeau M. Arahata K.. (1995). Absence of extraocular muscle pathology in Duchenne’s muscular dystrophy: role for calcium homeostasis in extraocular muscle sparing. J. Exp. Med. 182, 467–475. 10.1084/jem.182.2.467 7629506 PMC2192134

[B21] Latres E. Amini A. R. Amini A. A. Griffiths J. Martin F. J. Wei Y.. (2005). Insulin-like growth factor-1 (IGF-1) inversely regulates atrophy-induced genes via the phosphatidylinositol 3-kinase/Akt/mammalian target of rapamycin (PI3K/Akt/mTOR) pathway. J. Biol. Chem. 280, 2737–2744. 10.1074/jbc.m407517200 15550386

[B22] Lee S. J. Lee Y. S. Zimmers T. A. Soleimani A. Matzuk M. M. Tsuchida K.. (2010). Regulation of muscle mass by follistatin and activins. Mol. Endocrinol. 24, 1998–2008. 10.1210/me.2010-0127 20810712 PMC2954636

[B23] Lewis C. Ohlendieck K. (2010). Proteomic profiling of naturally protected extraocular muscles from the dystrophin-deficient mdx mouse. Biochem. Biophys. Res. Commun. 396, 1024–1029. 10.1016/j.bbrc.2010.05.052 20471957

[B24] McLoon L. K. Rowe J. Wirtschafter J. McCormick K. M. (2004). Continuous myofiber remodeling in uninjured extraocular myofibers: myonuclear turnover and evidence for apoptosis. Muscle Nerve 29, 707–715. 10.1002/mus.20012 15116375 PMC1796846

[B25] McLoon L. K. Thorstenson K. M. Solomon A. Lewis M. P. (2007). Myogenic precursor cells in craniofacial muscles. Oral Dis. 13, 134–140. 10.1111/j.1601-0825.2006.01353.x 17305613

[B26] McLoon L. K. Wirtschafter J. (2002a). Activated satellite cells are present in uninjured extraocular muscles of mature mice. Trans. Am. Ophthalmol. Soc. 100, 119–123; discussion 123–124. 12545684 PMC1358953

[B27] McLoon L. K. Wirtschafter J. D. (2002b). Continuous myonuclear addition to single extraocular myofibers in uninjured adult rabbits. Muscle Nerve 25, 348–358. 10.1002/mus.10056 11870711

[B28] McLoon L. K. Wirtschafter J. (2003). Activated satellite cells in extraocular muscles of normal adult monkeys and humans. Invest. Ophthalmol. Vis. Sci. 44, 1927–1932. 10.1167/iovs.02-0673 12714625 PMC1796845

[B29] Mitchell K. J. Pannérec A. Cadot B. Parlakian A. Besson V. Gomes E. R.. (2010). Identification and characterization of a non-satellite cell muscle resident progenitor during postnatal development. Nat. Cell Biol. 12, 257–266. 10.1038/ncb2025 20118923

[B30] Mozzetta C. Consalvi S. Saccone V. Tierney M. Diamantini A. Mitchell K. J.. (2013). Fibroadipogenic progenitors mediate the ability of HDAC inhibitors to promote regeneration in dystrophic muscles of young, but not old Mdx mice. EMBO Mol. Med. 5, 626–639. 10.1002/emmm.201202096 23505062 PMC3628105

[B31] Pacheco-Pinedo E. C. Budak M. T. Zeiger U. Jørgensen L. H. Bogdanovich S. Schrøder H. D.. (2009). Transcriptional and functional differences in stem cell populations isolated from extraocular and limb muscles. Physiol. Genomics 37, 35–42. 10.1152/physiolgenomics.00051.2008 19116248 PMC2661100

[B32] Pallafacchina G. François S. Regnault B. Czarny B. Dive V. Cumano A.. (2010). An adult tissue-specific stem cell in its niche: a gene profiling analysis of in vivo quiescent and activated muscle satellite cells. Stem Cell Res. 4, 77–91. 10.1016/j.scr.2009.10.003 19962952

[B33] Pannérec A. Formicola L. Besson V. Marazzi G. Sassoon D. A. (2013). Defining skeletal muscle resident progenitors and their cell fate potentials. Development 140, 2879–2891. 10.1242/dev.089326 23739133

[B34] Pannérec A. Marazzi G. Sassoon D. (2012). Stem cells in the hood: the skeletal muscle niche. Trends Mol. Med. 18, 599–606. 10.1016/j.molmed.2012.07.004 22877884

[B35] Partridge T. A. (2013). The mdx mouse model as a surrogate for Duchenne muscular dystrophy. FEBS J. 280, 4177–4186. 10.1111/febs.12267 23551987 PMC4147949

[B36] Porter J. D. (2002). Extraocular muscle: cellular adaptations for a diverse functional repertoire. Ann. N Y Acad. Sci. 956, 7–16. 10.1111/j.1749-6632.2002.tb02804.x 11960789

[B37] Porter J. D. Baker R. S. Ragusa R. J. Brueckner J. K. (1995). Extraocular muscles: basic and clinical aspects of structure and function. Surv. Ophthalmol. 39, 451–484. 10.1016/s0039-6257(05)80055-4 7660301

[B38] Porter J. D. Israel S. Gong B. Merriam A. P. Feuerman J. Khanna S.. (2006). Distinctive morphological and gene/protein expression signatures during myogenesis in novel cell lines from extraocular and hindlimb muscle. Physiol. Genomics 24, 264–275. 10.1152/physiolgenomics.00234.2004 16291736

[B39] Porter J. D. Khanna S. Kaminski H. J. Rao J. S. Merriam A. P. Richmonds C. R.. (2001). Extraocular muscle is defined by a fundamentally distinct gene expression profile. Proc. Natl. Acad. Sci. U S A 98, 12062–12067. 10.1073/pnas.211257298 11572940 PMC59827

[B40] Porter J. D. Merriam A. P. Khanna S. Andrade F. H. Richmonds C. R. Leahy P.. (2003). Constitutive properties, not molecular adaptations, mediate extraocular muscle sparing in dystrophic mdx mice. FASEB J. 17, 893–895. 12670877 10.1096/fj.02-0810fje

[B41] Relaix F. Weng X. Marazzi G. Yang E. Copeland N. Jenkins N.. (1996). Pw1, a novel zinc finger gene implicated in the myogenic and neuronal lineages. Dev. Biol. 177, 383–396. 10.1006/dbio.1996.0172 8806818

[B42] Relaix F. Zammit P. S. (2012). Satellite cells are essential for skeletal muscle regeneration: the cell on the edge returns centre stage. Development 139, 2845–2856. 10.1242/dev.069088 22833472

[B43] Sambasivan R. Gayraud-Morel B. Dumas G. Cimper C. Paisant S. Kelly R. G.. (2009). Distinct regulatory cascades govern extraocular and pharyngeal arch muscle progenitor cell fates. Dev. Cell 16, 810–821. 10.1016/j.devcel.2009.05.008 19531352

[B44] Sambasivan R. Kuratani S. Tajbakhsh S. (2011). An eye on the head: the development and evolution of craniofacial muscles. Development 138, 2401–2415. 10.1242/dev.040972 21610022

[B45] Sambasivan R. Tajbakhsh S. (2007). Skeletal muscle stem cell birth and properties. Semin. Cell Dev. Biol. 18, 870–882. 10.1016/j.semcdb.2007.09.013 18023213

[B46] Serrano A. L. Mann C. J. Vidal B. Ardite E. Perdiguero E. Muñoz-Cánoves P. (2011). Cellular and molecular mechanisms regulating fibrosis in skeletal muscle repair and disease. Curr. Top. Dev. Biol. 96, 167–201. 10.1016/b978-0-12-385940-2.00007-3 21621071

[B47] Shadrach J. L. Wagers A. J. (2011). Stem cells for skeletal muscle repair. Philos. Trans. R. Soc. Lond. B Biol. Sci. 366, 2297–2306. 10.1098/rstb.2011.0027 21727135 PMC3130421

[B48] Soltys J. Gong B. Kaminski H. J. Zhou Y. Kusner L. L. (2008). Extraocular muscle susceptibility to myasthenia gravis: unique immunological environment? Ann. N Y Acad. Sci. 1132, 220–224. 10.1196/annals.1405.037 18567871 PMC2527818

[B49] Stuelsatz P. Shearer A. Li Y. Muir L. A. Ieronimakis N. Shen Q. W.. (2014a). Extraocular muscle satellite cells are high performance myo-engines retaining efficient regenerative capacity in dystrophin deficiency. Dev. Biol. [Epub ahead of print]. 10.1016/j.ydbio.2014.08.035 25236433 PMC4309674

[B50] Stuelsatz P. Shearer A. Yablonka-Reuveni Z. (2014b). Ancestral Myf5 gene activity in periocular connective tissue identifies a subset of fibro/adipogenic progenitors but does not connote a myogenic origin. Dev. Biol. 385, 366–379. 10.1016/j.ydbio.2013.08.010 23969310 PMC3921074

[B51] Tabebordbar M. Wang E. T. Wagers A. J. (2013). Skeletal muscle degenerative diseases and strategies for therapeutic muscle repair. Annu. Rev. Pathol. 8, 441–475. 10.1146/annurev-pathol-011811-132450 23121053

[B52] Uezumi A. Fukada S. Yamamoto N. Takeda S. Tsuchida K. (2010). Mesenchymal progenitors distinct from satellite cells contribute to ectopic fat cell formation in skeletal muscle. Nat. Cell Biol. 12, 143–152. 10.1038/ncb2014 20081842

[B53] Uezumi A. Ito T. Morikawa D. Shimizu N. Yoneda T. Segawa M.. (2011). Fibrosis and adipogenesis originate from a common mesenchymal progenitor in skeletal muscle. J. Cell Sci. 124, 3654–3664. 10.1242/jcs.086629 22045730

[B54] Wallace G. Q. McNally E. M. (2009). Mechanisms of muscle degeneration, regeneration and repair in the muscular dystrophies. Annu. Rev. Physiol. 71, 37–57. 10.1146/annurev.physiol.010908.163216 18808326

[B55] Yin H. Price F. Rudnicki M. A. (2013). Satellite cells and the muscle stem cell niche. Physiol. Rev. 93, 23–67. 10.1152/physrev.00043.2011 23303905 PMC4073943

